# Isolated lesion of the oral mucosa

**DOI:** 10.1111/odi.14917

**Published:** 2024-08-23

**Authors:** Emmanuelle Vigarios, Serge Boulinguez, Béatrice Herbault‐Barres, Laurence Lamant, Saman Warnakulasuriya, Delphine Maret, Vincent Sibaud

**Affiliations:** ^1^ Department of Oral Medicine Institut Claudius Regaud, Institut Universitaire du Cancer Toulouse‐Oncopole Toulouse France; ^2^ Department of Dermatology Centre Hospitalo‐Universitaire de Toulouse, Hopital Larrey France Toulouse France; ^3^ Department of Oncodermatology Institut Claudius Regaud, Institut Universitaire du cancer Toulouse‐Oncopole Toulouse France; ^4^ Department of Pathology Institut Claudius Regaud, Institut Universitaire du Cancer Toulouse‐Oncopole Toulouse France; ^5^ The WHO Collaborating Centre, Oral Cancer and Faculty of Dentistry, Oral and Craniofacial Sciences King's College London London UK; ^6^ Faculté de Chirurgie Dentaire Université Paul Sabatier, Centre Hospitalier Universitaire Toulouse France

## 1 CASE REPORT

**FIGURE 1:**
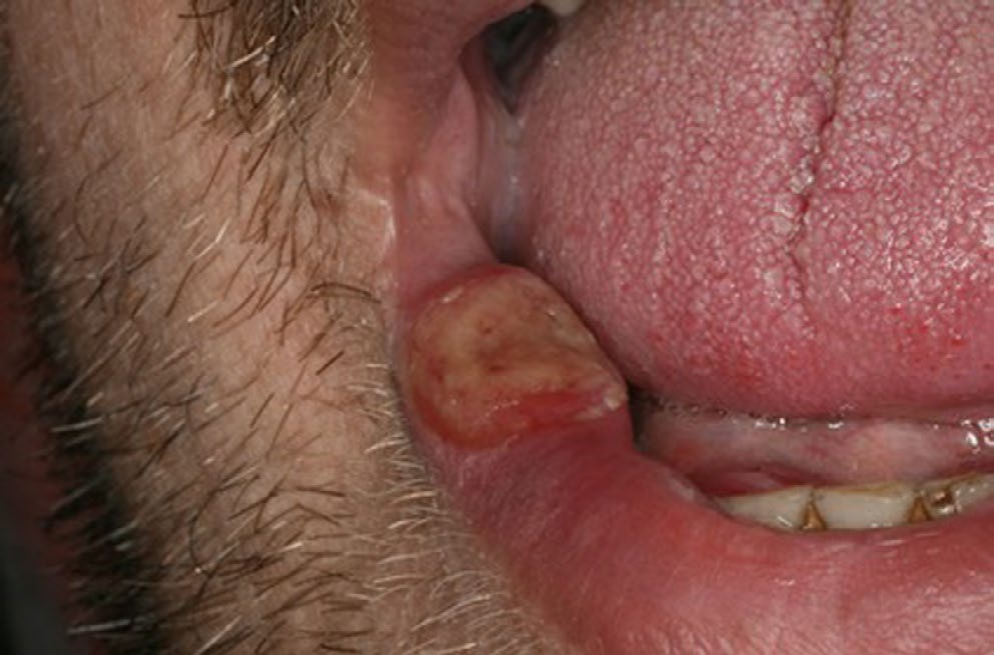
Solitary well demarcated necrotic ulcer located on the lower labial mucosa with raised and indurated margins.

A 34‐year‐old man presented to the Oral Medicine department with a 3‐week history of painful solitary ulcerated labial lesion that rapidly changed in size and color. He reported the development of two similar solitary lesions on the tongue 1 year before that had healed spontaneously within 3–4 weeks. No cutaneous or genital involvement was noticed in either of the flare‐ups. Oral examination revealed a well‐demarcated necrotic ulcer located on the lower labial mucosa with raised indurated margins (Figure 1). No personal history of dermatologic or systemic disease was noted. He had a 20‐pack‐year history of smoking. No cervical enlarged lymph nodes were present.

## 2 WHAT IS YOUR DIAGNOSIS?

Based on the patient's history and physical examinations, which one of the following is the most suspicious diagnosis?

- Lymphomatoid papulosis
- Granulomatosis with polyangiitis (Wegener)
- Squamous cell carcinoma
- Eosinophilic granuloma

## 3 DIAGNOSIS

**FIGURE 2:**
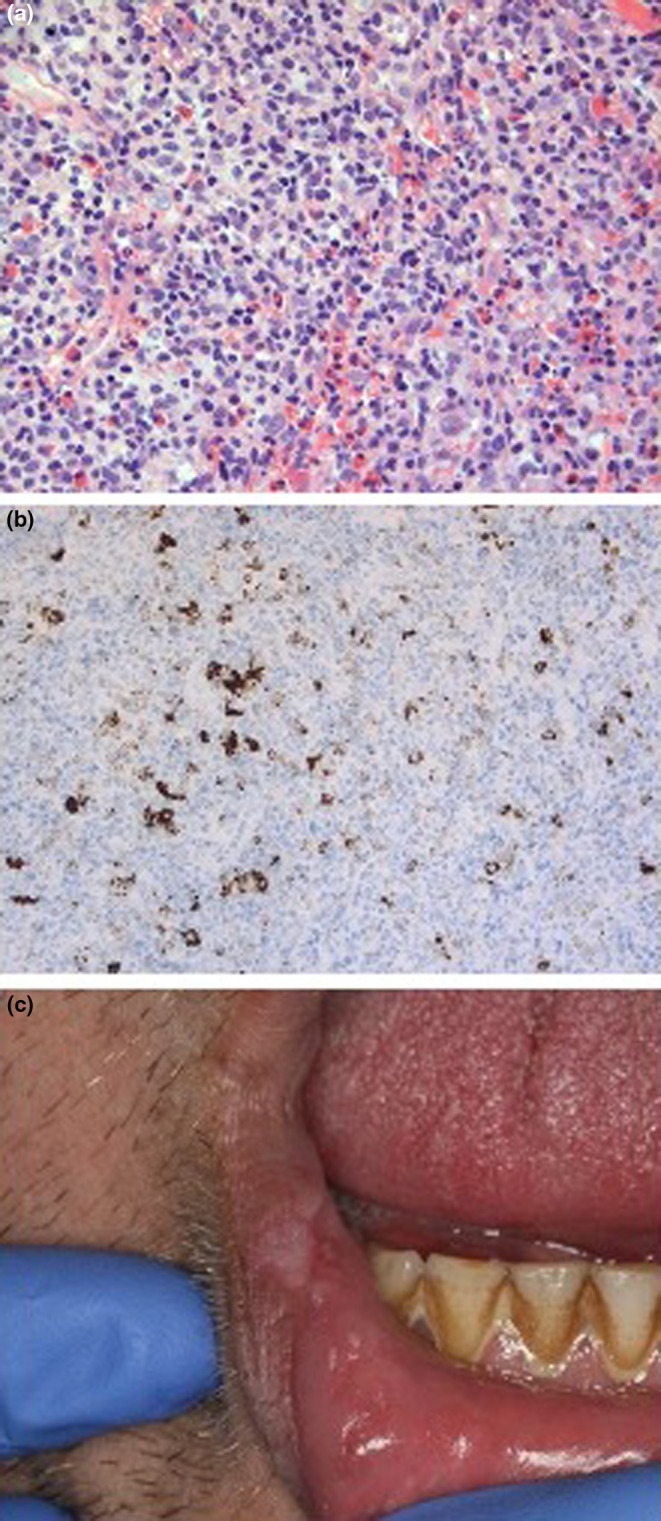
(a) Wedge‐shaped infiltrate consisting of scattered arranged medium‐to‐large atypical CD30+ lymphocytes admixed with histiocytes, eosinophils, and neutrophils (hematin‐eosin, original magnification 40); (b) scattered CD30+ cells demonstrated by immunohistochemistry (original magnification ×20); (c) spontaneous involution of necrotic lesion 3 weeks later. Scar secondary to incisional biopsy is visible.

The right diagnosis is A. An incisional mucosal biopsy was performed revealing acute ulceration surrounded by a dystrophic epithelium with no evidence of malignancy. Underlying connective and muscle showed a wedge‐shaped infiltrate consisting of scattered medium‐to‐large atypical lymphocytes admixed with histiocytes, eosinophils, and neutrophils (Figure 2a).

Immunohistochemical analysis revealed lymphoid cells of the CD3+ T‐phenotype and mostly of the CD4+ and CD5+ types. They displayed CD7 down‐regulation. Anti‐CD30 antibody showed isolated CD30+ T cells of medium size and immunoblastic morphology (Figure 2b). Anti‐ALK antibody was negative. Anti‐CD79a and anti‐CD20 antibodies revealed some reactive B lymphocytes. Anti‐PS100, CD1a and langerin antibodies identified some Langerhans cells of a reactive nature. Analysis of the T‐cell receptor (TCR) chain gene rearrangements revealed a predominantly clonal T‐cell population. Epstein–Barr virus‐encoded small nuclear RNA (EBER) analysis as well as fungal and bacterial swab cultures were all negative.

Based on the clinical history and histology a diagnosis of Lymphomatoid papulosis (LyP) was made.

LyP is an uncommon dermatologic disease that mainly occurs in adult male patients between the third and fourth decades (Nikolaenko et al., 2019). Children may occasionally experience it (de Misa et al., 2010; Nikolaenko et al., 2019). While CD30+ expression by lymphoid cells is a hallmarker of lymphocyte activation, lymphoproliferative disorders differ in their clinical presentation and histological features as well as their course, prognosis, and treatment (Kempf et al., 2018). LyP is a recurrent, chronic, self‐healing cutaneous condition characterized by several or multiple papulonodular or papulonecrotic lesions presenting histological abnormalities of malignant appearance (Allabert et al., 2008; Bretsztajn et al., 2019; de Misa et al., 2010; Kempf et al., 2011, 2018; Sciubba et al., 2000). Oral or genital involvement of LyP remains an uncommon event (Bretsztajn et al., 2019; de Misa et al., 2010; Kempf et al., 2018; Nikolaenko et al., 2019), particularly in the absence of associated cutaneous lesions. Most experts in the field consider as an indolent cutaneous T‐cell lymphoma on the same spectrum as primary cutaneous anaplastic large‐cell lymphoma (Hughey, 2015). In most cases, papulonodular lesions of LyP predominate on the trunk and limbs but can also occur on the face, scalp, palms, and soles as well as in the ano‐genital area (Allabert et al., 2008; Hughey, 2015; Kartan et al., 2019; Kempf et al., 2011, 2018; Martin et al., 2019; Nikolaenko et al., 2019). Oral lesions most commonly develop on the tongue (Bretsztajn et al., 2019), usually after the onset of cutaneous lesions. Conversely, oral lesions can sometimes occur several months to years before cutaneous involvement (Allabert et al., 2008).

The diagnosis of LyP of the oral mucosa is often difficult and complicated by the overlap in histomorphology between LyP and other reactive benign or malignant conditions (Sciubba et al., 2000).

Lymphomatoid papulosis can precede, follow, or be concomitant with another hematological malignancy. The diagnosis of lymphomatoid papulosis is important because it can lead to the early detection of another potentially dangerous hemopathy.

Despite the excellent prognosis of LyP which is a self‐healing disease, the recurrence of lesions or sometimes their location may require treatment (Hughey, 2015). To date, no curative treatment is available for LyP (Nikolaenko et al., 2019). However, high‐potency topical corticosteroids can be useful to alleviate pain and hasten the involution of mucosal lesions (Hughey, 2015; Kartan et al., 2019).

In the event of frequent outbreaks of many LyP lesions, various therapeutic approaches can be proposed including single‐agent systemic chemotherapy (etoposide, methotrexate), oral bexarotene, and/or phototherapy (Hughey, 2015; Nikolaenko et al., 2019). However, because of the benign course of the disease, the long‐term toxicity of these therapies should also be considered. To date, no data in the literature suggest that treatment of LyP lesions influences the natural history of the disease or reduces the risk of developing any associated lymphoproliferative disease (Allabert et al., 2008). Clinical appearance and clinical course over time are considered decisive criteria for the definite diagnosis (Kempf et al., 2018; Nikolaenko et al., 2019). Close communication between clinician and pathologist experienced in cutaneous lymphoma is mandatory in challenging cases (de Misa et al., 2010; Kempf et al., 2018).

## 4 OUTCOME

Over the 3 weeks after the biopsy, the lesion resolved spontaneously, only displaying a scar following the incisional biopsy (Figure 2c). Extra‐oral clinical examination, laboratory tests, and radiological examination consisting essentially of chest X‐ray, ultrasound of abdomen and pelvis, and CT scan did not provide any evidence for an associated lymphoma.

## AUTHOR CONTRIBUTIONS

**Emmanuelle Vigarios:** Conceptualization; data curation; writing – original draft; writing – review and editing. **Serge Boulinguez:** Conceptualization; writing – original draft; data curation; writing – review and editing. **Béatrice Herbault‐Barres:** Data curation; writing – review and editing. **Laurence Lamant:** Data curation; writing – review and editing. **Saman Warnakulasuriya:** Writing – review and editing. **Delphine Maret:** Writing – review and editing. **Vincent Sibaud:** Data curation; writing – review and editing.

## CONFLICT OF INTEREST STATEMENT

The authors do not declare any conflicts of interest related to this topic.

## PATIENT CONSENT

The patient reported in this manuscript provided written informed consent for the publication of the case details.

## Data Availability

Data sharing is not applicable to this article as no new data were created or analyzed in this study.
